# Supplementary material for: Hijacking of nucleotide biosynthesis and deamidation-mediated glycolysis by an oncogenic herpesvirus
Source: Nat Commun. 2024 Feb 16;15:1442. doi: 10.1038/s41467-024-45852-5 (PMC10873312; doi:10.1038/s41467-024-45852-5)
Supplement: Supplementary file 1 — Supplementary Information [file 41467_2024_45852_MOESM1_ESM.pdf]

Supplemental Figures for

Hijacking of Nucleotide Biosynthesis and Deamidation-mediated Glycolysis by an Oncogenic Herpesvirus

Quanyuan Wan[1], Leah Tavakoli[1], Ting-Yu Wang[2,3], Andrew J. Tucker[1], Ruiting Zhou[1], Qizhi Liu[2,6], Shu Feng[2,7], Dongwon Choi[4], Zhiheng He[5], Michaela U. Gack[1], and Jun Zhao[1*]

[1]Florida Research and Innovation Center, Cleveland Clinic, Port St. Lucie, FL, USA
[2]Section of Infection and Immunity, Herman Ostrow School of Dentistry, University of Southern California, Los Angeles, CA, USA
[3]Proteome Exploration Laboratory, Beckman Institute, California Institute of Technology, Pasadena, CA, USA
[4]Department of Surgery, Keck School of Medicine, University of Southern California, Los Angeles, CA, USA
[5]Department of Molecular Microbiology and Immunology, Keck School of Medicine, University of Southern California, Los Angeles, CA, USA
[6]Present address: State Laboratory of Developmental Biology of Freshwater Fish, Hunan Normal University, Changsha, Hunan, China
[7]Present address: Department of Diabetes & Cancer Metabolism, Beckman Research Institute of City of Hope, Duarte, CA, USA
*Corresponding author: zhaoj6@ccf.org

Fig S1. KSHV infection promotes CAD activity and induces RelA deamidation

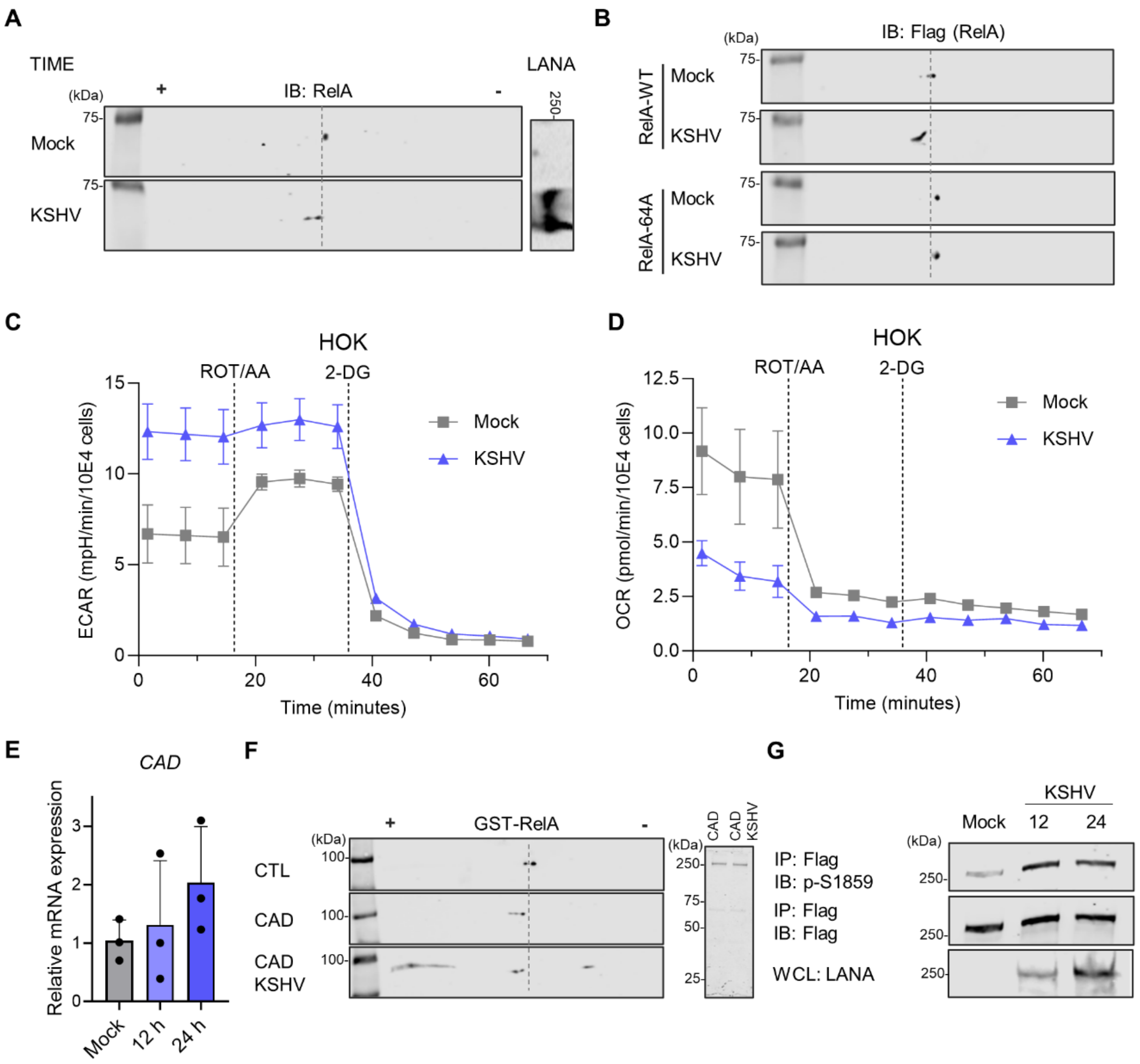

Figure S1. KSHV infection promotes CAD activity and induces RelA deamidation

(A) Tert-immortalized Microvascular Endothelial (TIME) cells were infected with KSHV (MOI = 3). Whole cell lysates (WCLs) were prepared at 48 h and analyzed by regular SDS-PAGE or two-dimensional gel electrophoresis (2DGE) and immunoblotting with anti-RelA and anti-LANA.
(B) Human Oral Keratinocytes-16B (HOKs) were reconstituted with Flag-tagged RelA wild-type (WT) or deamidation-resistant mutant (64A) by lentiviral transduction, and then infected with KSHV (MOI = 30). WCLs were prepared at 48 h and analyzed by 2DGE and immunoblotting with anti-Flag (RelA).
(C) HOKs were mock or infected with KSHV (MOI = 30) for 24 h. Extracellular acidification rate (ECAR) was measured by Seahorse XF mini.
(D) HOKs were mock or infected with KSHV (MOI = 30) for 24 h. Oxygen consumption rate (OCR) was measured by Seahorse XF mini.
(E) Real-time quantitative PCR (RT-qPCR) analysis of *CAD* mRNA in HOKs infected with KSHV (MOI = 30).
(F) 293T cells were transfected with a plasmid expressing Flag-tagged CAD. The transfected cells were then infected with KSHV (MOI = 5) for 24 h, before CAD was purified from mock and infected cells and analyzed by Coomassie staining (right panel). *In vitro* deamidation was performed with purified GST-RelA and analyzed by 2DGE and immunoblotting with anti-GST antibody (left panel).
(G) 293T cells transfected with plasmids expressing Flag-tagged CAD were infected with KSHV (MOI = 5) for the indicated hours. WCLs were precipitated with anti-Flag. Precipitated proteins and WCLs were analyzed by immunoblotting with the indicated antibodies.

Data are presented as mean ± SD (SEM for S1C and S1D) of n = 3 biological replicates (S1C-S1E). Blots were representative of at least two independent experiments (S1A, S1B, S1F). Source data are provided as a source data file.

Fig S2. KSHV vCyclin interacts with CAD and drives RelA deamidation

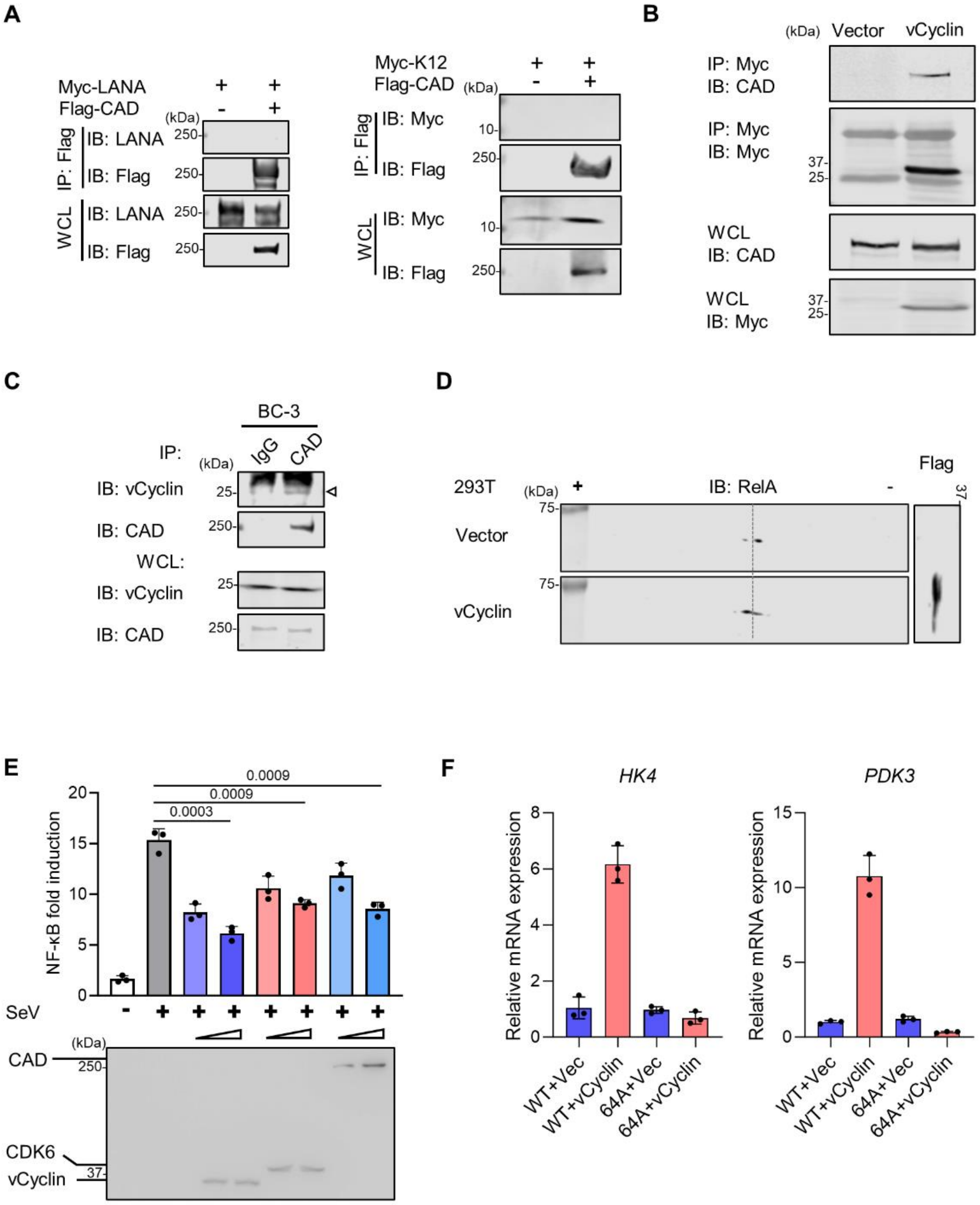

Figure S2. KSHV vCyclin interacts with CAD and drives RelA deamidation

(A) 293T cells were transfected with plasmids expressing Flag-CAD and Myc-KSHV latent proteins LANA (Left) or K12 (Right) for 48 h. WCLs were precipitated with anti-Flag (CAD). Precipitated proteins and WCLs were analyzed by immunoblotting with the indicated antibodies.
(B) 293T cells were transfected with plasmids expressing Myc-vCyclin for 48 h. WCLs were precipitated with anti-Myc. Precipitated proteins and WCLs were analyzed by immunoblotting with the indicated antibodies.
(C) WCLs from primary effusion lymphoma cell line BC-3 were precipitated with IgG or anti-CAD. Precipitated proteins and WCLs were analyzed by immunoblotting with the indicated antibodies.
(D) 293T cells were infected with lentivirus containing Flag-vCyclin. WCLs were prepared at 72 h and analyzed by regular SDS-PAGE or 2DGE and immunoblotting with anti-RelA and anti-Flag (vCyclin).
(E) 293T cells were transfected with the NF-κB reporter cocktail and increasing amounts of a plasmid containing vCyclin, CDK6, or CAD for 24 h. The transfected cells were then infected with Sendai virus (SeV, 100 HA U/ml) for 24 h and NF-κB activation was determined by luciferase assay.
(F) HOKs depleted of RelA by RelA-specific shRNA targeting 3'UTR were reconstituted with wild-type RelA (WT), or deamidation-resistant RelA (64A) by lentiviral transduction. The stable cells were then infected with lentivirus containing vCyclin. RT-qPCR analysis of *HK4* and *PDK3* mRNA was performed.

Data are presented as mean ± SD of n = 3 biological replicates (S2E and S2F). Blots were representative of at least two independent experiments (S2A-S2D). Significance was calculated using two-tailed, unpaired Student's t-test. Source data are provided as a source data file.

Fig S3. Metabolic reprogramming by KSHV vCyclin

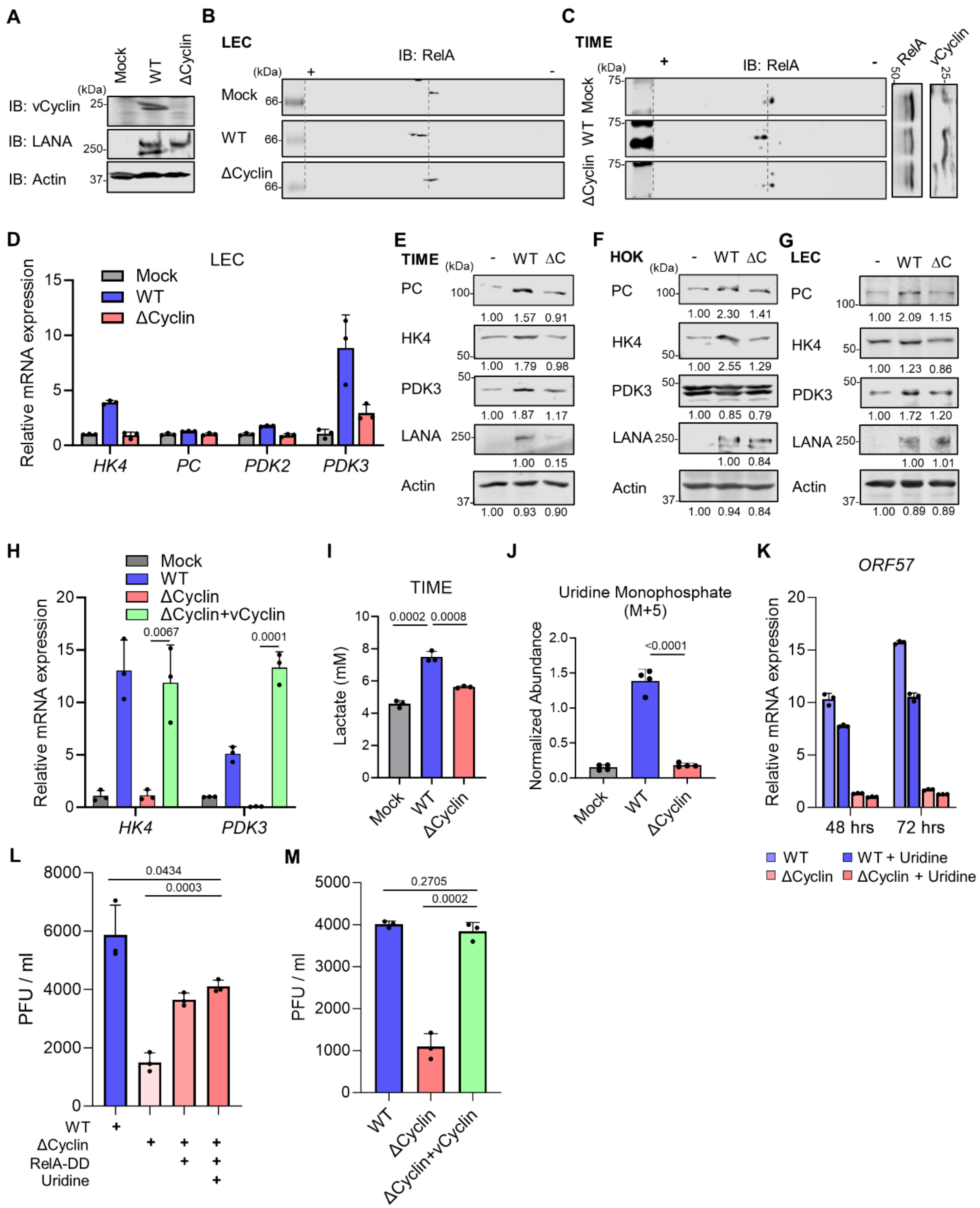

Figure S3. Metabolic reprogramming by KSHV vCyclin

(A) 293T cells were infected with KSHV-wild-type (WT) or KSHV-vCyclin.STOP (ΔCyclin) (MOI = 5) for 48 h. WCLs were analyzed by SDS-PAGE and immunoblotting with the indicated antibodies.
(B) LECs were infected with KSHV-WT or KSHV-ΔCyclin (MOI = 10) for 48 h. WCLs were analyzed by 2DGE and immunoblotting.
(C) TIME cells were infected with KSHV-WT or KSHV-ΔCyclin (MOI = 3) for 48 h. WCLs were analyzed by 2DGE and immunoblotting.
(D) RT-qPCR analysis of the indicated mRNAs in LECs infected with KSHV-WT or KSHV-ΔCyclin (MOI = 10) for 72 h.
(E) WCLs of infected TIME cells as in (C) were processed in parallel and analyzed by SDS-PAGE and immunoblotting with the indicated antibodies.
(F) HOKs were infected with KSHV-WT or KSHV-ΔCyclin (MOI = 30) for 48 h. The WCLs were processed in parallel and analyzed by immunoblotting with the indicated antibodies.
(G) WCLs of the infected LECs as in (B) were processed in parallel and analyzed by immunoblotting with the indicated antibodies.
(H) HOKs were transduced with lentivirus containing vector control or vCyclin, then infected with KSHV-WT or KSHV-ΔCyclin (MOI = 30) for 72 h. RT-qPCR analysis of the indicated mRNAs was performed.
(I) Lactate in the media of 1×10^6 infected TIME cells as in (C) at 16 h post medium replacement.
(J) Mass spectrometry analysis of uridine monophosphate (UMP) was performed at 15 min after labeling the HOKs pre-infected with KSHV-WT or KSHV-ΔCyclin. (M+5) indicates the mass of the labeled UMP with [U-^{13}C] glucose.
(K) RT-qPCR analysis of *ORF57* mRNA in HOKs infected with KSHV-WT or KSHV-ΔCyclin (MOI = 30) for the indicated hours with or without uridine (10 µg/ml).
(L) HOKs were depleted of RelA by RelA-specific shRNA targeting 3'UTR and then reconstituted with RelA-DD. The reconstituted cells were then infected with KSHV-WT or KSHV-ΔCyclin (MOI = 30) for 72 h with or without uridine (10 µg/ml). Viral titers in the culture medium were determined.
(M) The viral titers in the media of the infected HOKs as in (H).

Data are presented as mean ± SD of n = 3 biological replicates (S3D, S3H, S3I, S3K-S3M) and n = 4 biological replicates (S3J). Blots were representative of at least two independent experiments (S3A-S3C, S3E-S3G). Significance was calculated using two-tailed, unpaired Student's t-test. Source data are provided as a source data file.

Fig S4. CDK6 phosphorylates CAD at S1900

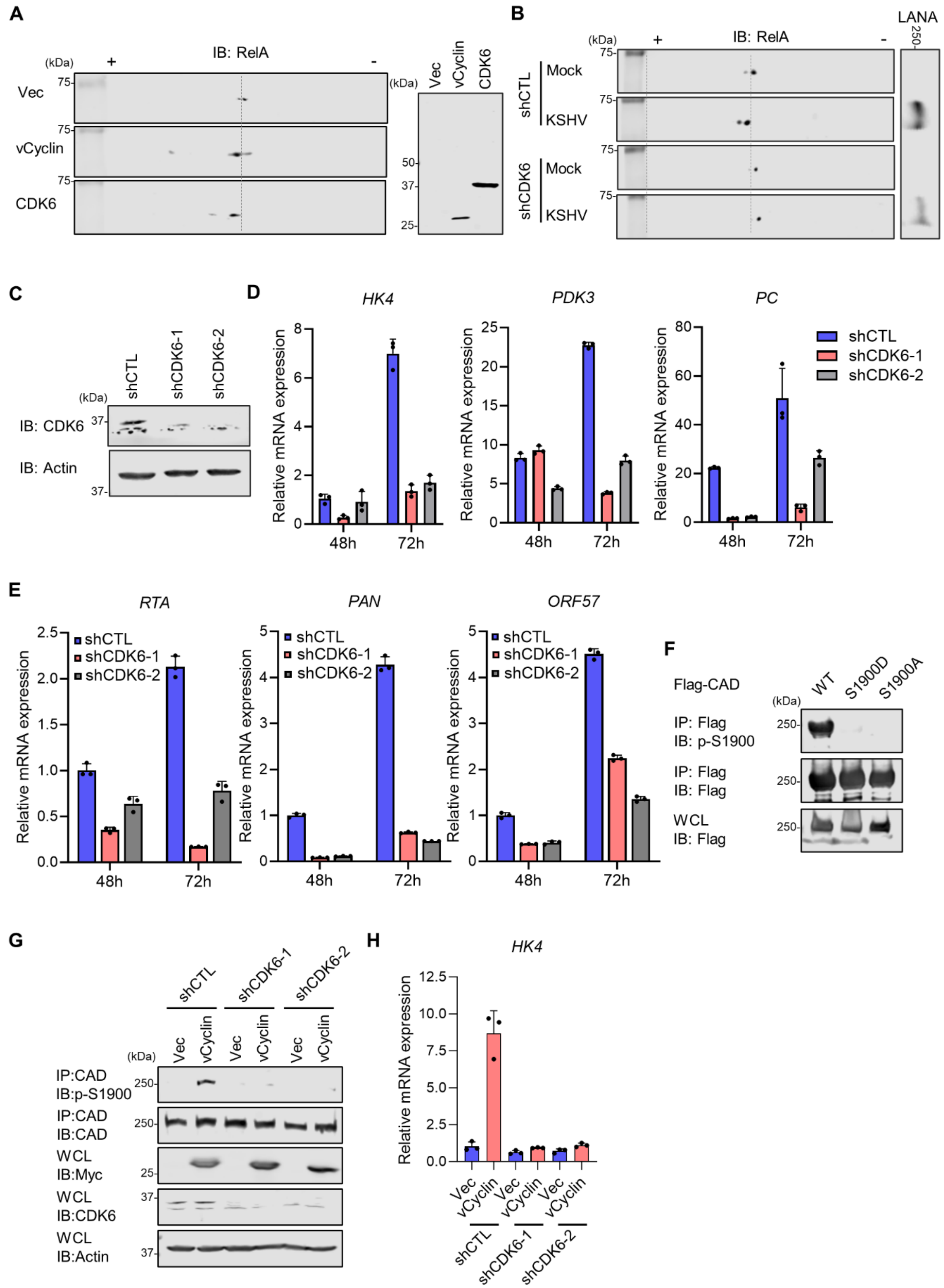

Figure S4. CDK6 phosphorylates CAD at S1900

(A) 293T cells were transfected with plasmids expressing vCyclin or CDK6 for 48 h. WCLs were analyzed by regular SDS-PAGE or two-dimensional gel electrophoresis (2DGE) and immunoblotting with anti-RelA, anti-Myc (vCyclin), and anti-Flag (CDK6).
(B) HOKs depleted of CDK6 by shRNA were infected with KSHV (MOI = 30) for 48 h. WCLs were analyzed by regular SDS-PAGE or 2DGE and immunoblotting with anti-RelA and anti-LANA.
(C) HOKs were depleted of CDK6 by two CDK6-specific shRNAs. WCLs were analyzed by immunoblotting with the indicated antibodies.
(D) RT-qPCR analysis of the indicated mRNAs in HOKs depleted of CDK6 as in (C) and infected with KSHV (MOI = 30) for the indicated hours.
(E) RT-qPCR analysis of the indicated viral mRNAs in HOKs depleted of CDK6 as in (C) and infected with KSHV (MOI = 30) for the indicated hours.
(F) 293T cells were transfected with plasmids expressing Flag-CAD-WT, S1900D, or S1900A for 48 h. WCLs were precipitated with anti-Flag (CAD). Precipitated proteins and WCLs were analyzed by immunoblotting with the indicated antibodies.
(G) 293T cells were depleted of CDK6 by CDK6-speific shRNA and then transfected with empty vector or Myc-vCyclin for 48 h. WCLs were precipitated with anti-CAD. Precipitated proteins and WCLs were analyzed by immunoblotting with the indicated antibodies.
(H) RT-qPCR analysis of *HK4* mRNA in HOKs depleted of CDK6 by CDK6-speific shRNA and infected with lentivirus containing vector control or vCyclin for 48 h.

Data are presented as mean ± SD of n = 3 biological replicates (S4D, S4E, and S4H). Blots were representative of at least two independent experiments (S4A, S4B, S4F). Source data are provided as a source data file.

Fig S5. CAD S1900 phosphorylation promotes metabolic reprogramming

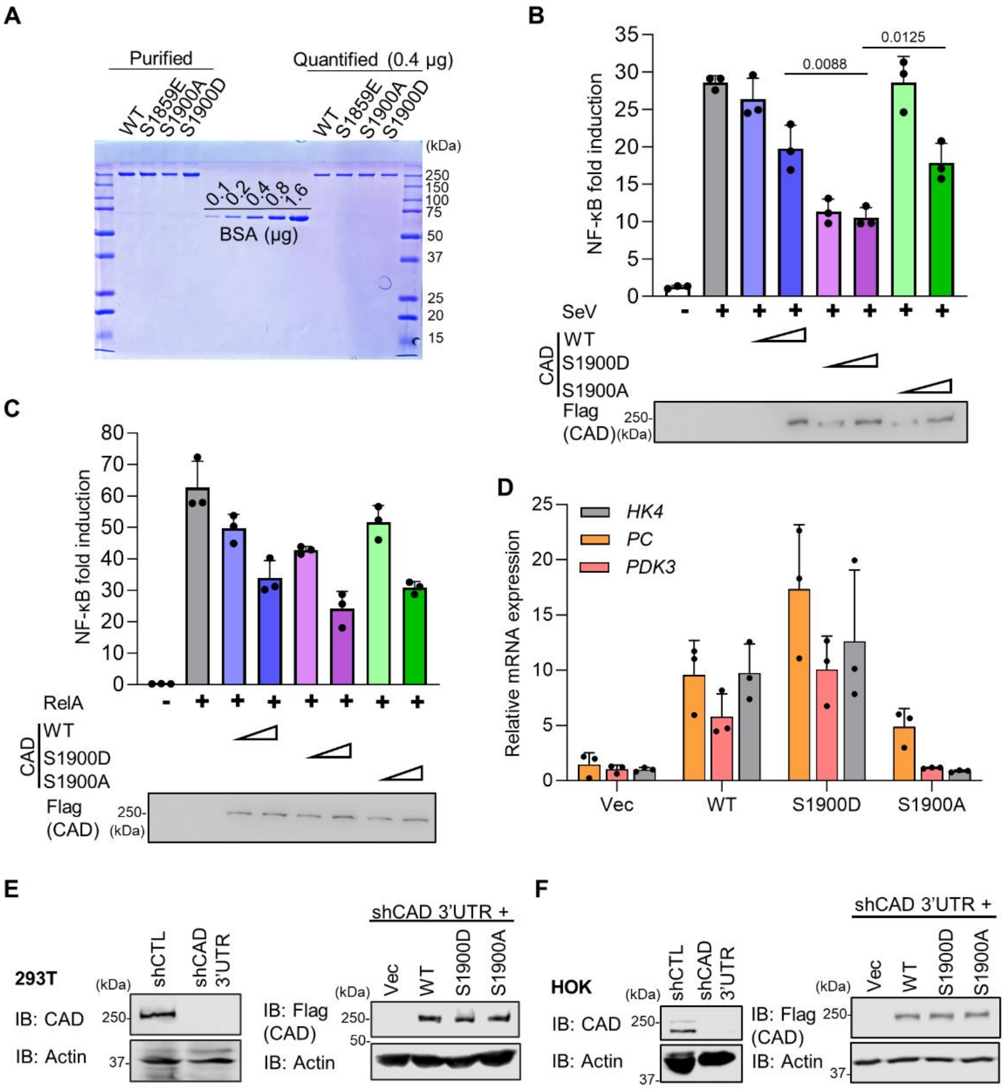

Figure S5. CAD S1900 phosphorylation promotes metabolic reprogramming

(A) 293T cells were transfected with plasmids expressing CAD wild-type or the indicated mutants for 48 h. CAD proteins were purified, eluted, analyzed by SDS-PAGE, and stained with Coomassie blue.
(B) 293T cells were transfected with the NF-κB reporter cocktail and plasmids containing CAD mutants for 24 h. The transfected cells were then infected with Sendai virus (SeV, 100 HA U/ml) for 24 h and NF-κB activation was determined by luciferase assay.
(C) 293T cells were transfected with the NF-κB reporter cocktail and plasmids containing RelA together with CAD mutants for 30 h. NF-κB activation was determined by luciferase assay.
(D) RT-qPCR analysis of the indicated mRNAs in $CAD^{-/-}$ 293T cells reconstituted with CAD WT, S1900D, or S1900A by transient transfection for 48 h.
(E) 293T cells were depleted of CAD by CAD-specific shRNA targeting 3'UTR and then reconstituted with the indicated CAD mutants by transient transfection. WCLs were analyzed by immunoblotting with the indicated antibodies.
(F) HOKs were depleted of CAD by CAD-specific shRNA targeting 3'UTR and then reconstituted with the indicated CAD mutants by transient transfection. WCLs were analyzed by immunoblotting with the indicated antibodies.

Data are presented as mean ± SD of n = 3 biological replicates (S5B-S5D). Blots were representative of at least two independent experiments (S5A). Significance was calculated using two-tailed, unpaired Student's t-test. Source data are provided as a source data file.

Fig S6. CAD and RelA Deamidation for KSHV pathogenesis

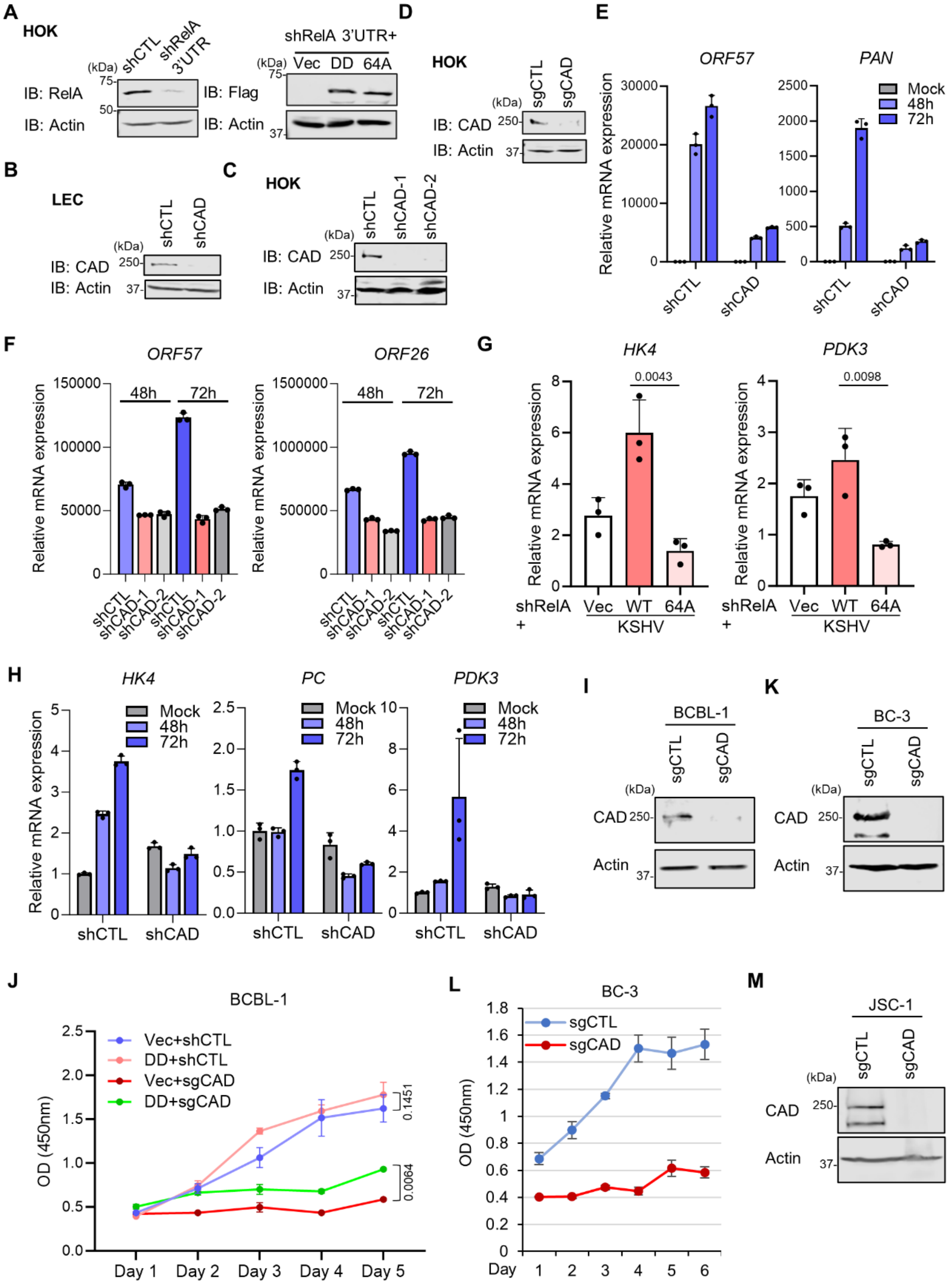

Figure S6. CAD and RelA deamidation for KSHV pathogenesis

(A) HOKs were depleted of RelA by RelA-specific shRNA targeting 3'UTR and then reconstituted with RelA deamidation mutants by lentiviral transduction. WCLs were processed in parallel and analyzed by immunoblotting with the indicated antibodies.
(B) LECs were depleted of CAD by CAD-specific shRNA. WCLs were analyzed by immunoblotting with the indicated antibodies.
(C) HOKs were depleted of CAD by CAD-specific shRNAs. WCLs were analyzed by immunoblotting with the indicated antibodies.
(D) HOKs were depleted of CAD by CRISPR-Cas9/sgCAD. WCLs were analyzed by immunoblotting with the indicated antibodies.
(E) RT-qPCR analysis of the indicated viral mRNAs in LECs depleted of CAD and infected with KSHV.
(F) RT-qPCR analysis of the indicated viral mRNAs in HOKs depleted of CAD and infected with KSHV.
(G) TIME cells were depleted of RelA by RelA-specific shRNA targeting 3'UTR and then reconstituted with RelA-WT or RelA-64A by lentiviral transduction. RT-qPCR analysis of the indicated mRNAs was then performed for the reconstituted cells infected with KSHV (MOI = 3) for 48 h.
(H) RT-qPCR analysis of the indicated mRNAs in LECs depleted of CAD and infected with KSHV.
(I) WCLs of BCBL-1 cells depleted of CAD by CRISPR-Cas9/sgCAD were analyzed by immunoblotting with the indicated antibodies.
(J) BCBL-1 cells were reconstituted with RelA-DD by lentiviral transduction. The stable cell lines were then depleted of CAD by CRISPR-Cas9/sgCAD. Cell proliferation was determined for 5 days.
(K) WCLs of BC-3 cells depleted of CAD by CRISPR-Cas9/sgCAD were analyzed by immunoblotting with the indicated antibodies.
(L) Proliferation of BC-3 cells as in (K) for 6 days.
(M) WCLs of JSC-1 cells depleted of CAD by CRISPR-Cas9/sgCAD were analyzed by immunoblotting with the indicated antibodies.

Data are presented as mean ± SD of n = 3 biological replicates (S6E-S6H, S6J, and S6L). Blots were representative of at least two independent experiments (S6I, S6K, S6M). Significance was calculated using two-tailed, unpaired Student's t-test (paired t-test for S6J). Source data are provided as a source data file.

Fig S7. CDK6 and CAD inhibitors block KSHV pathogenesis

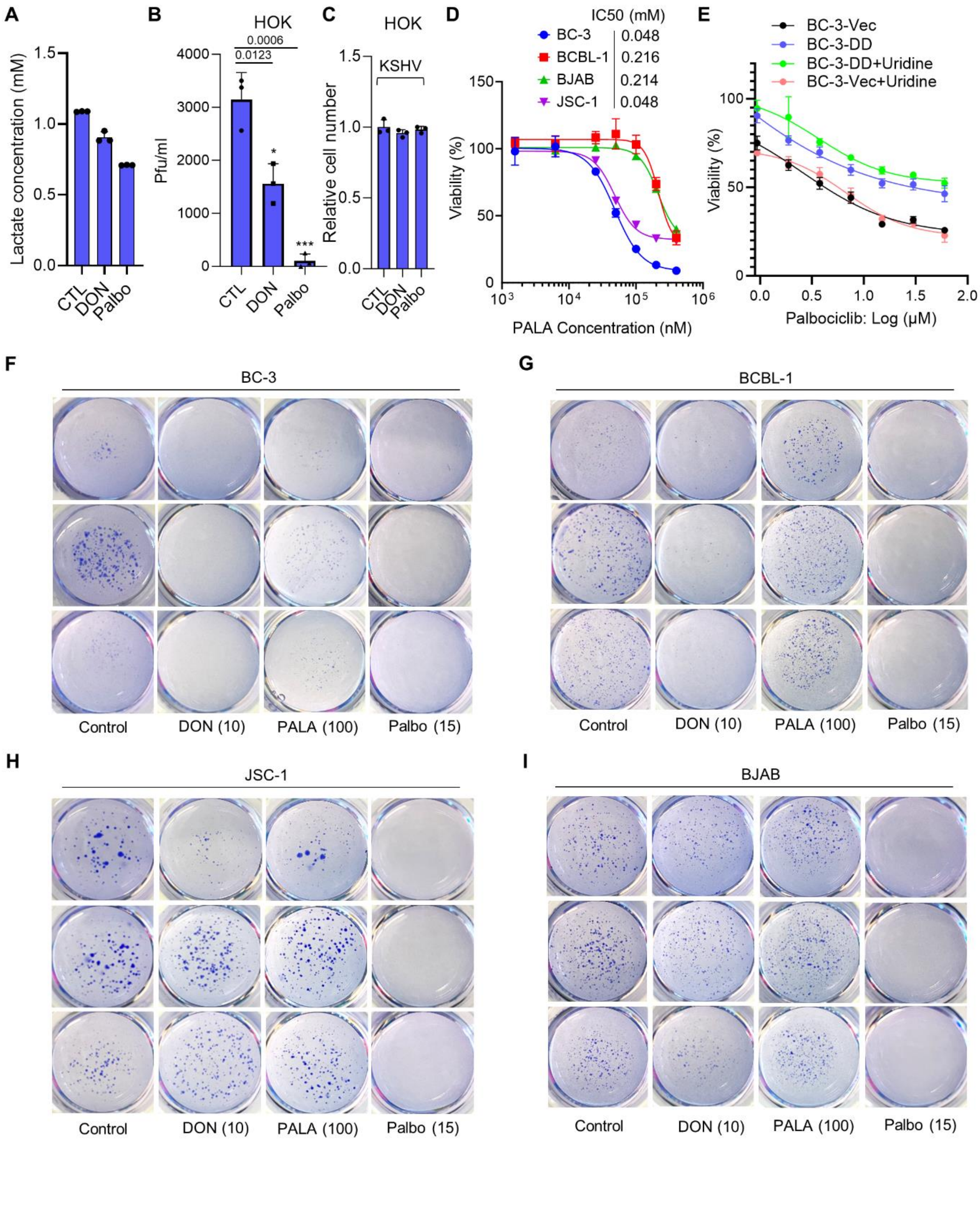

Figure S7. CDK6 and CAD inhibitors block KSHV pathogenesis

(A) Lactate in the media of the LECs treated with DON (30 µM) or Palbociclib (2 µM) for 72 h.
(B) Viral titers in the media of HOKs infected with KSHV (MOI = 30) for 24 h and then treated with DON (30 µM) or Palbociclib (5 µM) for 48 h.
(C) Relative cell numbers of the HOKs infected with KSHV (MOI = 30) for 24 h and then treated with DON (30 µM) or Palbociclib (5 µM) for 48 h.
(D) Viability of BC-3, BCBL-1, BJAB, and JSC-1 cells in the presence of increasing concentrations of N-phosphonacetyl-l-aspartate (PALA).
(E) Viability of RelA-DD-reconstituted BC-3 cells with or without uridine (10 µg/ml) in the presence of increasing concentrations of Palbociclib.
(F) Colony formation assay of BC-3 cells in the presence of DON (10 µM), PALA (100 µM), or Palbociclib (15 µM).
(G) Colony formation assay of BCBL-1 cells in the presence of DON (10 µM), PALA (100 µM), or Palbociclib (15 µM).
(H) Colony formation assay of JSC-1 cells in the presence of DON (10 µM), PALA (100 µM), or Palbociclib (15 µM).
(I) Colony formation assay of BJAB cells in the presence of DON (10 µM), PALA (100 µM), or Palbociclib (15 µM).

Data are presented as mean ± SD of n = 3 biological replicates (S7A-S7E). Significance was calculated using two-tailed, unpaired Student’s t-test. Source data are provided as a source data file.

Fig S8. CDK6 and CAD inhibitors block KSHV pathogenesis

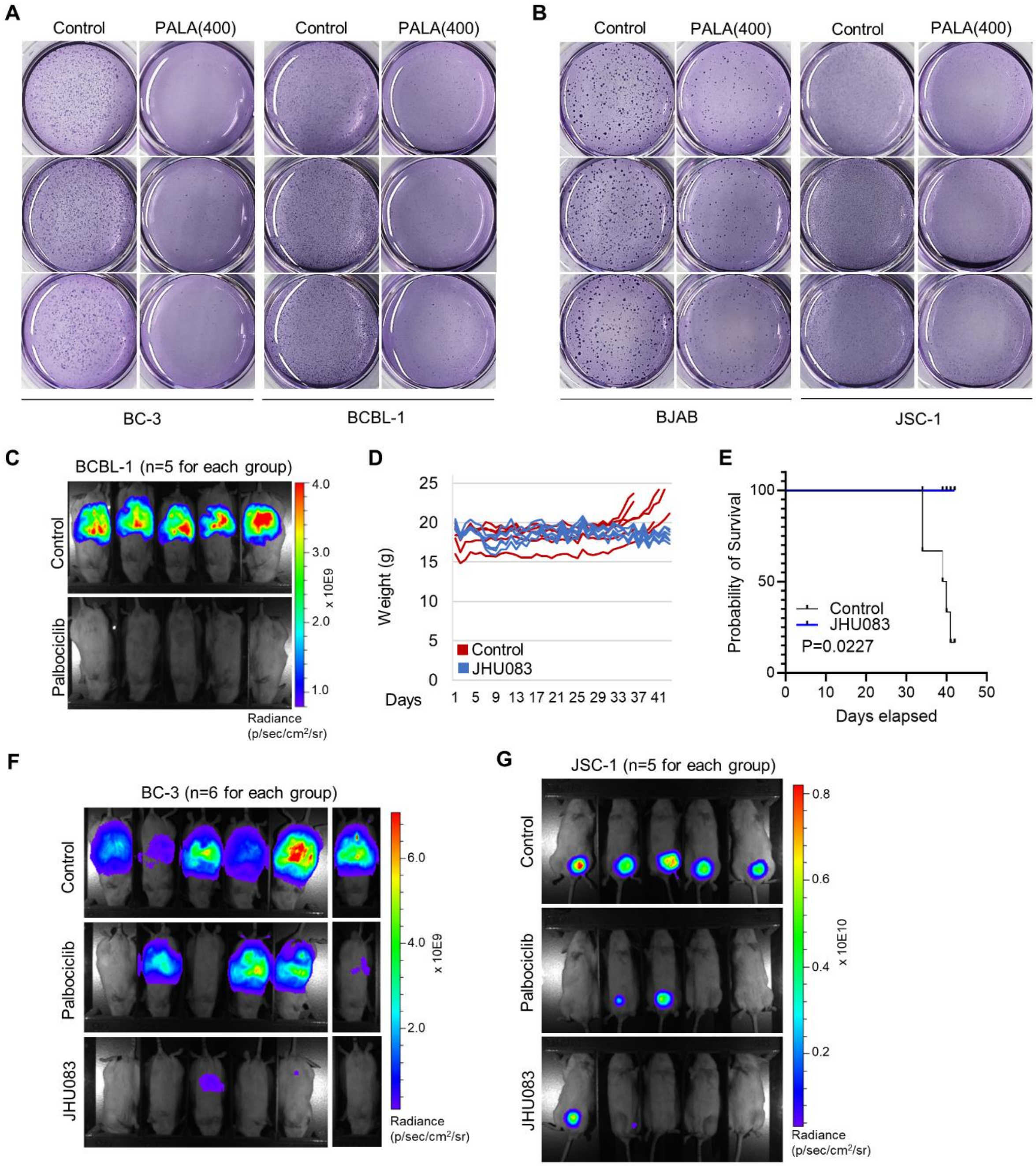

Figure S8. CDK6 and CAD inhibitors block KSHV pathogenesis

(A) Colony formation assay of BC-3 and BCBL-1 cells in the presence of PALA (400 μM).
(B) Colony formation assay of BJAB and JSC-1 cells in the presence of PALA (400 μM).
(C) IVIS imaging of the BCBL-1-luciferase-engrafted NOD-SCID mice at day 21 post treatment with Palbociclib.
(D) Mice weights of the BCBL-1-luciferase-engrafted NOD-SCID mice treated with JHU083.
(E) Survival curve of the BCBL-1-luciferase-engrafted NOD-SCID mice treated with JHU083.
(F) IVIS imaging of the BC-3-luciferase-engrafted NOD-SCID mice treated with Palbociclib or JHU083.
(G) IVIS imaging of the JSC-1-luciferase-engrafted NOD-SCID mice treated with Palbociclib or JHU083.

Source data are provided as a source data file.

Supplementary Table 1. RT-qPCR primers for this study

Human β-actin	
Forward	5’-CTGGCACCCAGCACAATG-3’
Reverse	5’-GCCGATCCACACGGAGTACT-3’
Human CAD	
Forward	5’-TGCTCACCTATCCTCTGATCG-3’
Reverse	5’-GCTGGGAGTAGGACAGCAC-3’
Human PDK2	
Forward	5’-CCGCTGTCCATGAAGCAGTT-3’
Reverse	5’-TGCCTGAGGAAGGTGAAGGA-3’
Human PDK3	
Forward	5'-CAAGCAGATCGAGCGCTACTC-3'
Reverse	5'-CGAAGTCCAGGAATTGTTTGATG-3'
Human HK4	
Forward	5’-GCTTGTGATTCTGGGATGGA-3’
Reverse	5’-GCTATGGGAGCTGAAGATGTAG-3’
Human PC	
Forward	5’-GATAGTGTCTGCCTTCTGGAGAGC-3’
Reverse	5’-ACACACGGATGGCAATCTCACC-3’
KSHV K8.1	
Forward	5’-CACCACAGAACTGACCGATG-3’
Reverse	5’-TGGCACACGGTTACTAGCAC-3’
KSHV LANA	
Forward	5’-TTGGATCTCGTCTTCCATCC-3’
Reverse	5’-ACCAGACGATGACCCACAAC-3’
KSHV ORF26	
Forward	5’-TATTCTGCAGCAGCTGTTGG-3’
Reverse	5’-TCTACGTCCAGACGATATGTGC-3’
KSHV ORF57	
Forward	5’-TGCTCTTGGCCTTTGTCCTA-3’
Reverse	5’-TGCACAAGCTGTGATGTTCC-3’
KSHV PAN	
Forward	5’-CCGCCGATTGTGGGTTGATT-3’
Reverse	5’-TTTTGTTCTGCGGGCTTATGGAG-3’
KSHV RTA	
Forward	5’-GAGTCCGGCACACTGTACC-3’
Reverse	5’-AAACTGCCTGGGAAGTTAACG-3’
