# Supplementary material for: Hijacking of nucleotide biosynthesis and deamidation-mediated glycolysis by an oncogenic herpesvirus
Source: Nat Commun. 2024 Feb 16;15:1442. doi: 10.1038/s41467-024-45852-5 (PMC10873312; doi:10.1038/s41467-024-45852-5)
Supplement: Supplementary file 3 — Reporting Summary [file 41467_2024_45852_MOESM3_ESM.pdf]

nature portfolio

Corresponding author(s): Jun Zhao

Last updated by author(s): Jan 22, 2024

Reporting Summary

Nature Portfolio wishes to improve the reproducibility of the work that we publish. This form provides structure for consistency and transparency in reporting. For further information on Nature Portfolio policies, see our Editorial Policies and the Editorial Policy Checklist.

Statistics

For all statistical analyses, confirm that the following items are present in the figure legend, table legend, main text, or Methods section.

n/a	Confirmed	
☐	☒	The exact sample size (*n*) for each experimental group/condition, given as a discrete number and unit of measurement
☐	☒	A statement on whether measurements were taken from distinct samples or whether the same sample was measured repeatedly
☐	☒	The statistical test(s) used AND whether they are one- or two-sided<br>*Only common tests should be described solely by name; describe more complex techniques in the Methods section.*
☒	☐	A description of all covariates tested
☒	☐	A description of any assumptions or corrections, such as tests of normality and adjustment for multiple comparisons
☐	☒	A full description of the statistical parameters including central tendency (e.g. means) or other basic estimates (e.g. regression coefficient) AND variation (e.g. standard deviation) or associated estimates of uncertainty (e.g. confidence intervals)
☐	☒	For null hypothesis testing, the test statistic (e.g. *F*, *t*, *r*) with confidence intervals, effect sizes, degrees of freedom and *P* value noted<br>*Give P values as exact values whenever suitable.*
☒	☐	For Bayesian analysis, information on the choice of priors and Markov chain Monte Carlo settings
☒	☐	For hierarchical and complex designs, identification of the appropriate level for tests and full reporting of outcomes
☒	☐	Estimates of effect sizes (e.g. Cohen's *d*, Pearson's *r*), indicating how they were calculated

Our web collection on statistics for biologists contains articles on many of the points above.

Software and code

Policy information about availability of computer code

Data collection	LI-COR Image Studio, Bio-Rad CFX Manager, BioTek GEN5, PelkinElmer Living Image, Seahorse XF HS Mini Controller, Amersham Typhoon Control Software, Q Exactive Plus Mass Spectrometer Software
Data analysis	MS Excel, GraphPad Prism 10, LI-COR Image Studio, Bio-Rad CFX Manager, Proteome Discoverer 2.4, Interactive Peptide Spectral Annotator, TraceFinder 4.1, Agilent Seahorse Analytics, R (IsoCorrectoR package)

For manuscripts utilizing custom algorithms or software that are central to the research but not yet described in published literature, software must be made available to editors and reviewers. We strongly encourage code deposition in a community repository (e.g. GitHub). See the Nature Portfolio guidelines for submitting code & software for further information.

Data

Policy information about availability of data

All manuscripts must include a data availability statement. This statement should provide the following information, where applicable:

- Accession codes, unique identifiers, or web links for publicly available datasets
- A description of any restrictions on data availability
- For clinical datasets or third party data, please ensure that the statement adheres to our policy

The mass spectrometry proteomics data have been deposited to the ProteomeXchange Consortium via the PRIDE partner repository with the dataset identifier

PXD043435. All data needed to evaluate the conclusions for the study are present in the paper. Unique expression vectors and reagents generated for this study are available upon request. Source data are provided with this paper.

Research involving human participants, their data, or biological material

Policy information about studies with human participants or human data. See also policy information about sex, gender (identity/presentation), and sexual orientation and race, ethnicity and racism.

Reporting on sex and gender	n/a
Reporting on race, ethnicity, or other socially relevant groupings	n/a
Population characteristics	n/a
Recruitment	n/a
Ethics oversight	n/a

Note that full information on the approval of the study protocol must also be provided in the manuscript.

Field-specific reporting

Please select the one below that is the best fit for your research. If you are not sure, read the appropriate sections before making your selection.

☒ Life sciences ☐ Behavioural & social sciences ☐ Ecological, evolutionary & environmental sciences

For a reference copy of the document with all sections, see nature.com/documents/nr-reporting-summary-flat.pdf

Life sciences study design

All studies must disclose on these points even when the disclosure is negative.

Sample size	Sample sizes were determined by power analyses for in vivo studies. The numbers of replicates were chosen based on standard practices. In general, experiments were performed with three or four biological replicates where applicable. Details of each experiment were included in the figure legends.
Data exclusions	No exclusion.
Replication	Two or three replications with similar results were performed for key experiments. See details in respective figure legends.
Randomization	Not applicable to in vitro experiments. For in vivo experiments, mice were randomized into vehicle and therapeutic groups.
Blinding	Not applicable to the study. No human participant is involved in the study.

Reporting for specific materials, systems and methods

We require information from authors about some types of materials, experimental systems and methods used in many studies. Here, indicate whether each material, system or method listed is relevant to your study. If you are not sure if a list item applies to your research, read the appropriate section before selecting a response.

Materials & experimental systems

n/a	Involved in the study	
☐	☒	Antibodies
☐	☒	Eukaryotic cell lines
☒	☐	Palaeontology and archaeology
☐	☒	Animals and other organisms
☒	☐	Clinical data
☒	☐	Dual use research of concern
☒	☐	Plants

Methods

n/a	Involved in the study	
☒	☐	ChIP-seq
☒	☐	Flow cytometry
☒	☐	MRI-based neuroimaging

Antibodies

Antibodies used	Antibodies against GST (Z-5, B-14, Santa Cruz), FLAG (M2, Sigma), V5 (D3H8Q, Cell Signaling), CAD (A301-374A, Bethyl Laboratories), RelA (51-0500, Thermofisher), Pyruvate Carboxylase (66470, Cell Signaling, 49381, Cell Signaling), PDK3 (AP7040a, Abcepta), Hexokinase 4 (AP7901c, Abcepta), β-Actin (8H10D10, Cell Signaling), Phospho-CAD (Ser1859, Cell Signaling), LANA (LN53, Abcam), vCyclin (94B, Abcam), MYC(71D10, 9B11, Cell Signaling), CDK6 (D4S8S, Cell Signaling) were purchased from the indicated suppliers. Polyclonal antibodies against KSHV vCyclin and Phospho-CAD (Ser1900) were generated by Genscript.
Validation	All commercial antibodies were purchased from vendors with validation and citations provided on their websites for the use of Western Blotting or Immunoprecipitation (IP). Antibodies against KSHV vCyclin and Phospho-CAD (Ser1900) were validated by the Zhao laboratory and the results were included in the manuscript.

Eukaryotic cell lines

Policy information about cell lines and Sex and Gender in Research

Cell line source(s)	HEK293T was purchased from ATCC. HOK16B, iSLK.BAC16-WT, iSLK.BAC16-deltaCyclin, TIME, LEC, BCBL-1, BC-3, JSC-1, and BJAB were obtained from other investigators as described in the Methods section of the manuscript.
Authentication	Cells from ATCC were authenticated by the vendor. Cell lines which were obtained from and validated by other investigators were not further authenticated. ISLK.BAC16-WT and iSLK.BAC.16.deltaCyclin cell lines were validated by KSHV production and immunoblotting for vCyclin expression upon KSHV infection. The presence of KSHV in BCBL-1, BC-3, and JSC-1, as well as the absence of KSHV in BJAB were confirmed by RT-qPCR or immunoblotting.
Mycoplasma contamination	All cell lines for this study were tested negative for potential mycoplasma contamination by PCR-based Mycoplasma detection assays.
Commonly misidentified lines (See ICLAC register)	iSLK.BAC16 cell lines (derived from Caki-1) were only used for KSHV production.

Animals and other research organisms

Policy information about studies involving animals; ARRIVE guidelines recommended for reporting animal research, and Sex and Gender in Research

Laboratory animals	Six to fourteen weeks NOD.Cg-Prkdcscid/J (NOD-SCID, The Jackson Laboratory) mice were used for all tumor xenograft experiments. All mice were housed in a standard pathogen-free animal facility.
Wild animals	No wild animals were used in the study.
Reporting on sex	Female mice were used for the xenograft experiments.
Field-collected samples	No field collected samples were used in the study.
Ethics oversight	All animal work was performed under strict accordance with the recommendation in the Guide for the Care and Use of Laboratory Animals of the National Institutes of Health. The protocol was approved by the Institutional Animal Care and Use Committee (IACUC) of the Cleveland Clinic FRIC.

Note that full information on the approval of the study protocol must also be provided in the manuscript.
